# Could screw/hook insertion at the apical vertebrae with rib head dislocation effectively retract the corresponding rib head from spinal canal in dystrophic scoliosis secondary to type 1 neurofibromatosis?

**DOI:** 10.1186/s12891-022-05248-2

**Published:** 2022-03-25

**Authors:** Song Li, Saihu Mao, Yanyu Ma, Ben-long Shi, Zhen Liu, Ze-zhang Zhu, Jun Qiao, Yong Qiu

**Affiliations:** Division of Spine Surgery, Department of Orthopedic Surgery, Nanjing Drum Tower Hospital, The Affiliated Hospital of Nanjing University Medical School, Nanjing, Jiangsu Province, China

**Keywords:** Dystrophic scoliosis, NF1, Rib head dislocation, Screw/hook insertion

## Abstract

**Background:**

Rib head dislocation (RHD) in dystrophic scoliosis of type 1 neurofibromatosis (DS-NF1) is a unique disorder caused by skeletal dystrophy and scoliotic instability. No particular surgical manipulation is mentioned in the literature to instruct the spine surgeons to effectively obtain more migration of the dislocated rib head without resection. The present study aimed to investigate the effectiveness of screw/hook insertion at vertebrae with RHDs on the retraction of penetrated rib head from spinal canal.

**Methods:**

37 neurologically intact patients with DS-NF1 and concomitant 53 RHDs undergoing scoliosis surgery without rib head excision were retrospectively reviewed. We used pre and postoperative whole-spine radiographs to determine the Cobb angle and the vertebral translation (VT), and the CT scans to evaluate the intraspinal rib length (IRL) and rib-vertebral angle (RVA). The dislocated ribs were assigned into two groups according to the presence of screw/hook insertion at vertebrae with RHD: screw/hook group and non-screw/hook group.

**Results:**

37 dislocated ribs with screws/hooks insertion at corresponding vertebrae were assigned into the screw/hook group and the remaining 16 dislocated ribs consisted of the non-screw/hook group. In the screw/hook group, the correction rates of Cobb angle and VT were significantly higher than the non-screw/hook group after surgery (58.7 ± 16.0% vs. 30.9 ± 12.4%, *p* = 0.003; 61.8 ± 18.8% vs. 35.1 ± 16.6%, *p* = 0.001; respectively). Similarly, more correction rates of IRL and RVA were found in the screw/hook group than the non-screw/hook group (63.1 ± 31.3% vs. 30.1 ± 20.7%, *p* = 0.008; 17.6 ± 9.7% vs. 7.2 ± 3.6%, *p* = 0.006; respectively). Multiple linear regression analysis revealed that the correction rates of Cobb angle, VT and RVA contributed significantly to correction of IRL (β = 0.389, 0.939 and 1.869, respectively; *p* = 0.019, 0.001 and 0.002, respectively).

**Conclusion:**

Screw/hook insertion at dystrophic vertebrae with RHDs contributed significantly to the degree of retraction of penetrated rib head from spinal canal. This effectiveness is mediated by more corrections of VT and RVA.

## Introduction

Rib head dislocation (RHD) and invasion into the spinal canal is not a rare characteristic of dystrophic scoliosis secondary to type 1 neurofibromatosis (DS-NF1), with an incidence ranging from 12.1–15.9% in the literatures [1–6]. The potential risk of cord injury and the painful rib hump by palpation are two unique characteristics of this phenomenon [5, 7]. Resection of the compressing rib head has been advocated for patients with consequential neurological impairments caused by RHD [2, 4, 6, 8, 9]. Those with an increasing occurrence of a provoked shock-like painful rib hump under direct pressure are also candidates for rib head resection. Contrarily, for majority of such patients who are neurologically intact, current consensus proposes that preservation of the dislocated rib head is not a contraindication to deformity correction [5, 8, 10]. Spontaneous migration of rib head from spinal canal has been well reported following scoliosis correction with modern spinal instrumentation techniques [5, 8, 10]. In this situation, a rib excision is not desirable yet may be applied as preventive strategies for neurological impairments, if necessary.

Having been well documented in previous reports, RHD uniformly occur on the convex side of the scoliosis and was combined with rib penciling [11], enlargement of foramen and significant vertebral rotation to the convexity in the apical region among DS-NF1 patients [5, 12, 13]. Thus, theoretically, corrective vertebral translation and derotation to the concavity are beneficial for retracting the penetrated rib head from spinal canal, which is confirmed in the previous reports [5, 8, 10]. Yalcin et al. reported that, under the direct visualization, the penetrated rib head migrated significantly from the canal, and meanwhile, the apex moved to the concavity during correction maneuvers [8]. Mao et al. found that the extraction degree of rib head from the spinal canal was associated with the correction of vertebral translation and rib-vertebrae angle immediately after the surgery [5]. However, no particular surgical manipulation was mentioned in the literature to instruct the spine surgeons to effectively obtain more pull-out of the embedded rib head without rib head resection. Usually, adequate apical screw/hook placement along with correction maneuvers can achieve significant reduction of vertebral translation and desired vertebral derotation in idiopathic scoliosis [14, 15]. For dystrophic scoliosis secondary to NF-1, this concept is supposed to be applicable as well. However, adequate apical screw placement is a big challenge for NF-1 patients even with the assistance of O-arm-based navigation techniques. Screw misplacement due to pedicle dystrophy may cause devastating neurological injury and increasing risk of screw poor pull-out strength, which renders risk/benefit assessment a dilemma for this situation.

Currently, no quantitative analysis was performed regarding whether screw/hook placement in the vertebrae with RHD could be an optimal strategy for retracting the dislocated rib head from the spinal canal. This study is designed to amend this issue, so as to provide detailed data needed to determine whether or not to the fixation placement in dystrophic pedicle is beneficial and should be tried with utmost effort in case of RHD with impending neurological deficits.

### Patients and methods

#### Patients

**Fig. 1:**
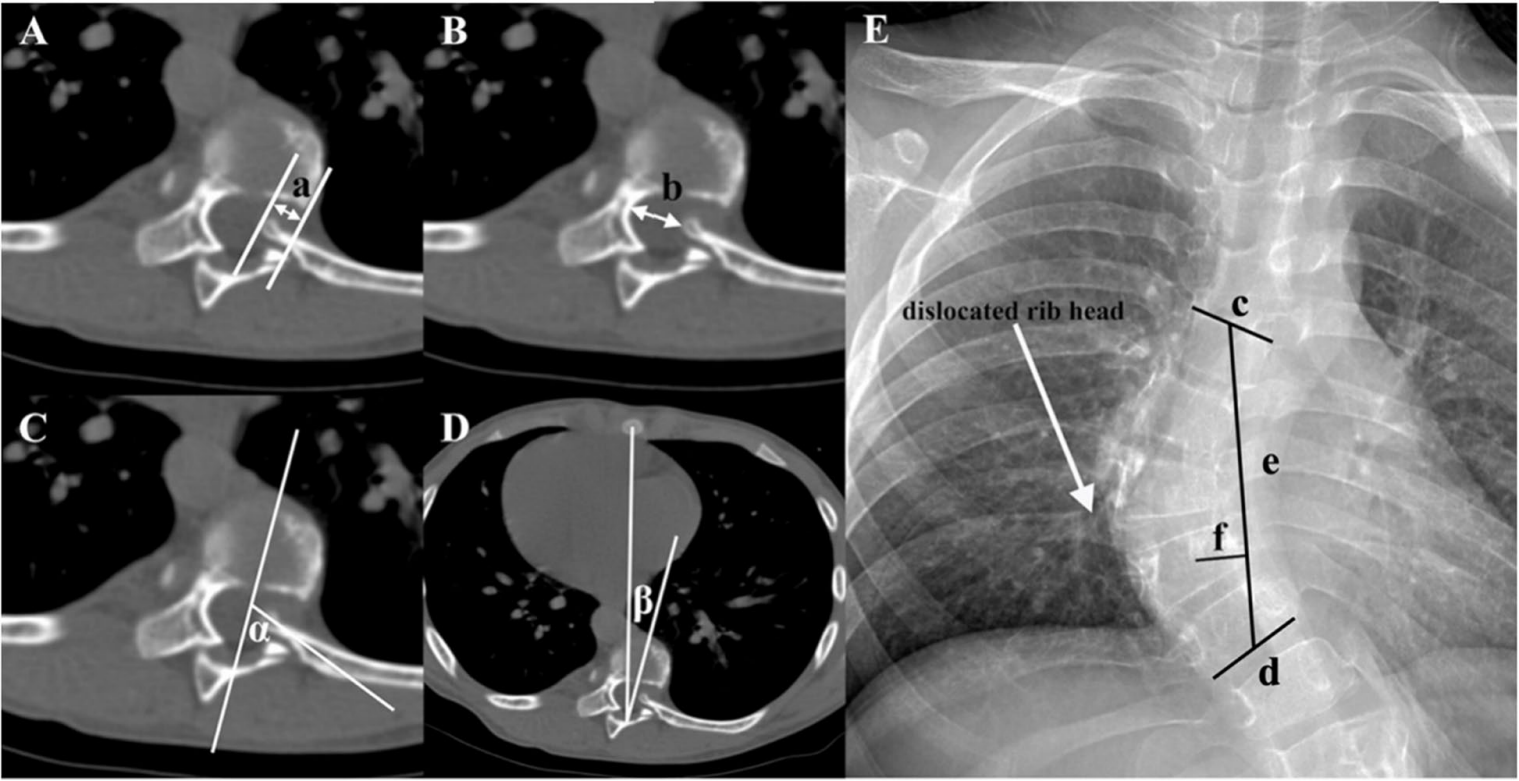
Illustration of the measurements of the intraspinal rib length (IRL), the distance between the rib head tip and the most concave spot of the osseous spinal canal (DRCSSC), the rib-vertebral angle (RVA), the vertebral-sternum angle, the spinal height and the vertebral translation. **A** IRL (line a). **B** DRCSSC (line b). **C** RVA (α). **D** Vertebral-sternum angle (β). **E** The height of main curve (line e) and the vertebral translation (line f). c and d are the lengths of the superior endplate of the upper end vertebra of the main curve and the inferior endplate of the lower end vertebra of the main curve, respectively. Line e is defined as the height of main curve which is formed by the midpoints of line c and d. Line f is the vertebral translation presenting the centrum of vertebra with dislocated rib head translating away from line e

**Fig. 2:**
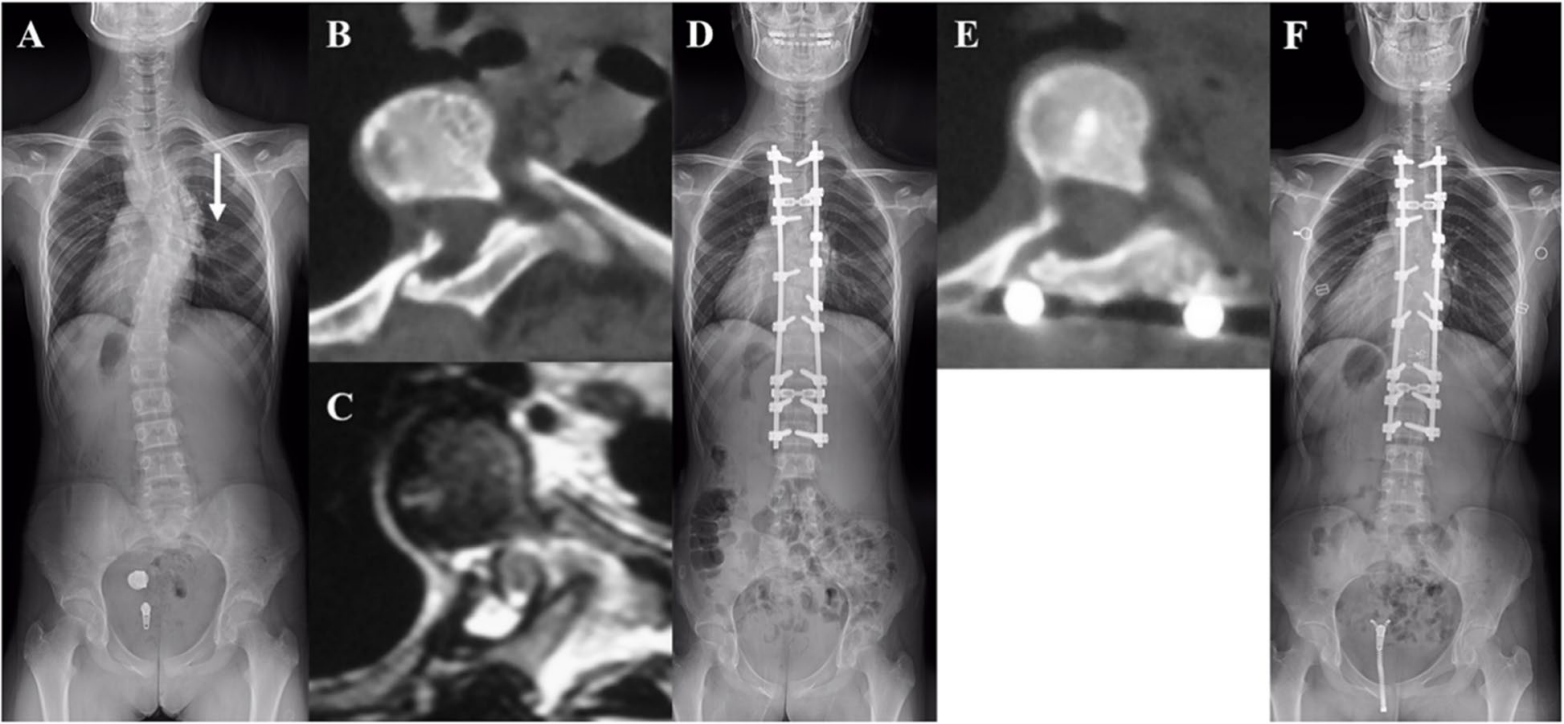
A 13-year-old girl with right thoracic dystrophic scoliosis secondary to NF-1 received surgical treatment in our institution. **A** The preoperative Cobb angle of thoracic curve was 55° with 7^th^ rib head dislocation and the vertebral translation (VT) of T7 was 28.5 mm **B **and **C**. The preoperative CT and MRI scans revealed the 7^th^ rib head penetrating the foramen and compressing the dural sac but not the spinal cord. The intraspinal rib length (IRL) reached 10.3 mm and the T7 showed a notable rib-vertebral angle (RVA) of 70°. **D** Posterior-only spinal fusion without rib head excision was performed, and pedicle screw was inserted at the level of dislocated rib. The Cobb angle was corrected to 24° postoperatively. **E** A good degree of rib head withdrawal was confirmed by post-operative CT scans. The IRL, RVA and VT were corrected to 2.8 mm, 57° and 13.5 mm postoperatively

**Fig. 3:**
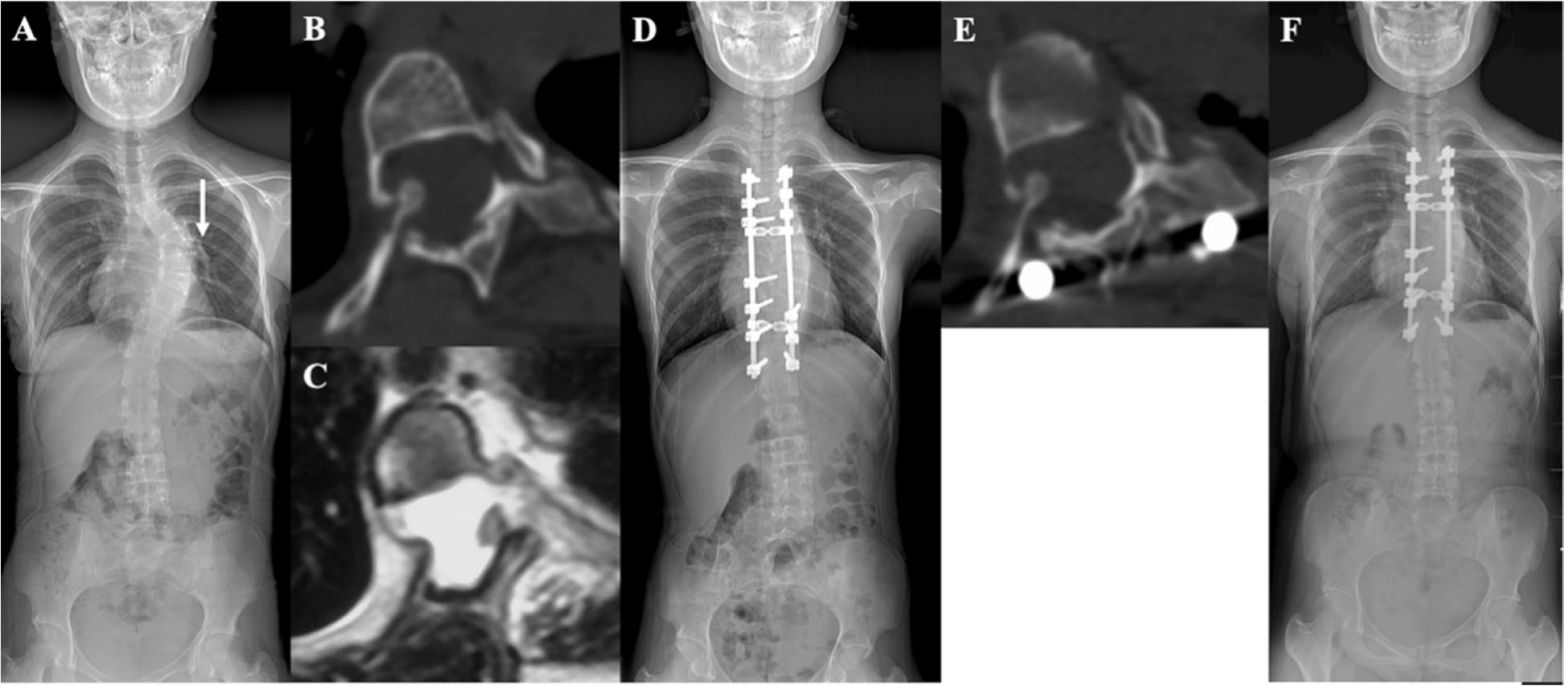
A 14-year-old boy with left thoracic dystrophic scoliosis secondary to NF-1 received surgical treatment in our institution. **A** The preoperative Cobb angle of thoracic curve was 60° with 8^th^ rib head dislocation (white arrow) and the VT of T8 was 12.2 mm **B **and** C**. Preoperative CT and MRI scans revealed the 8^th^ rib head penetrating the foramen and compressing the dural sac but not the spinal cord. The IRL and the VRA were 12.2 mm and 75.3°, respectively. **D** Posterior-only spinal fusion without rib head excision was performed, and pedicle screw or hook was not placed at the level of dislocated rib head. The Cobb angle was corrected to 31° postoperatively. **E** A minor degree of rib head withdrawal was confirmed by postoperative CT scans. The IRL, RVA and VT were corrected to 9.9 mm, 69.6° and 12.4 mm postoperatively

This is a retrospective study approved by the Institution Review Board of our hospital. Patients with dystrophic scoliosis secondary to NF-1 who underwent corrective scoliosis surgery between March 1998 to January 2018 were identified from our scoliosis database. The following inclusion criteria were used: (1) thoracic scoliosis with typical dystrophic radiographic features [1]; (2) rib head penetrating into the spinal canal identified on preoperative CT scans (Fig. 1); (3) both pre- and postoperative images of axial CT scans. Exclusion criteria were as follows: (1) preoperative intra-canal neurofibromatosis; (2) combined with preoperative neurological impairments; (3) treated with growing rods; (4) history of spine surgery. The medical records, imaging scans, and operative reports were reviewed. Patient demographic information was recorded including age at surgery, sex, curve pattern, vertebral level of RHD, surgical strategies, fusion levels, postoperative neurological status, and surgical complications. Patients were assigned into two groups according to whether or not there existed apical fixation at the vertebrae with RHD: screw/hook group (Fig. 2) and non-screw/hook group (Fig. 3).

### Radiographic assessment

Radiographic analysis included the Cobb angle in both coronal and sagittal planes on long-cassette standing posteroanterior and lateral radiographs obtained preoperatively, early postoperatively (within two weeks). The CT scans were used to analyze and assess the variations of the positional rib-canal interrelationships caused by corrective maneuvers. The parameters for positional assessments were measured pre- and post-operatively as follows: (1) intraspinal rib length [5]; (2) the distance between the rib head tip and the most concave spot of the spinal canal (DRCSSC) [5]; (3) rib-vertebrae angle; (4) vertebral-sternum angle [16]; (5) height of main curve; (6) vertebral translation. The definitions of the standard measuring techniques of the aforementioned parameters were as follows.

- 1. Intraspinal rib length (IRL, Fig. 1A): length of the penetrated rib head measured from the rib head tip to the intersection point of rib head with the border of the spinal canal [5].
- 2. DRCSSC (Fig. 1B): distance between the rib head tip and the most concave point of the osseous spinal canal, which was the point where the deviated spinal cord rested in a distorted and rotated spine canal with apparent lateral deviation [5].
- 3. Rib-vertebrae angle (RVA, Fig. 1C): defined as the angle between the axis of the rib head and the line bisecting the vertebral body longitudinally.
- 4. Vertebral-sternum angle (Fig. 1D): defined as the angle between the line bisecting the vertebral body longitudinally and the line drawn from the middle point of the sternum to the dorsal central aspect of the vertebral foramen [16].
- 5. Height of main curve (Fig. 1E): distance between the two midpoints of the superior and inferior endplates of the corresponding upper and lower end vertebrae of the main curve.
- 6. Vertebral translation (Fig. 1E): vertical distance from the center point of the vertebra with RHD to the line defined as the height of main curve.

Parameters were measured on axial CT scans (parameters 1–4) or the standing posteroanterior radiographs (parameters 5 and 6) using the PACS (Picture Archiving and Communications Systems, PACS) workstation (Easy Vision IDS5, version 11.4, Philips, Hamburg, Germany). Pre- and postoperative CT scans in supine position were obtained in all patients, which were performed preoperatively to provide anatomical information on the severity of the deformed bony vertebral structure to guide the selection and insertion of appropriate pedicle screws, and postoperatively to detect any mispositioned pedicle screws potentially causing neural or vascular injury or impingement. All the patients and their parents were informed about the purpose of CT scans and the potential risks, who then signed informed consent forms. The selection of measurement level on CT scan was identified as the rib head tip with maximal rib penetration and intrusion at the level of intervertebral foramina, both pre- and post-operatively. Two of the well-trained authors (S.L. and Y.Y.M.) completed the measurement individually. In addition, all parameters were selected to determine the inter- and intra- observer variability of the measurement. All the radiographic parameters were measured by the authors and then repeated twice. There were strong inter-observer and intra-observer agreements for all the parameters with all the kappa correlation coefficients exceeding 0.8. Therefore, the measured data were highly reliable, and the mean values of the parameters measured by the two investigators were recorded.

### Statistical analysis

Data analysis was conducted using SPSS 19.0 software (IBM). Shapiro–Wilk test was firstly used to check for the normal distribution of data before performing the *t* test. Normal distribution test revealed that all *p* values were > 0.05 in preoperative and postoperative data, or data from groups with or without screw/hook insertion, which indicated that all the data included for the *t* test were within the normal distribution. Paired-sample *t* test was applied to compare the operative changes in each group. In addition, the data were compared between the groups with the use of independent-sample *t*. Multiple linear regression analysis was used to explore whether correction of Cobb angle, vertebral translation, and rib-vertebrae angle could contribute to the extraction of intra-canal rib heads. The significance level was < 0.05.

## Results

### Demographic data

Totally 37 patients (20 females, 54%) with a mean age of 14.8 ± 5.3 years (range, 8–33 years) at surgery were recruited. The intraspinal RHD uniformly located on the convexity of the curve apex regions. Single-level RHD accounted for the largest share (23, 62%), followed by double RHD (12, 32%) and triple RHD (2, 6%). Accordingly, 53 dislocated rib heads were finally recruited and analyzed, and their location was categorized as apex-1 level for 10 cases (19%), apex level for 23 cases (43%), apex + 1 level for 19 cases (36%), and apex + 2 level for 1 case (2%) (Table 1). All patients were surgically treated by three experienced senior surgeons (Y.Q., Z.L. and Z.Z.Z.). Posterior-only spinal fusion with all pedicle screw or hybrid constructs was employed in all patients. One patient received Smith-Peterson osteotomy to correct the regional kyphosis. No monitoring alert in somatosensory evoked potential (SEP) or motor evoked potential (MEP) was encountered during surgery.

**Table 1:** Clinical information of the recruited NF1 patients with rib head dislocations

	**(Mean ± SD)/no**
**Sex (F/M)**	20/17
**Age (y)**	14.8 ± 5.3 (range, 8–33)
**Curve type**	
Right thoracic curve	16
Left thoracic curve	4
Double thoracic curve	9
Double major curve	8
**Apex location**	
T4	1 (2.7%)
T5	10 (27%)
T6	11 (29.7%)
T7	5 (13.5%)
T8	4 (10.8%)
T9	4 (10.8%)
T10	1 (2.7%)
T11	1 (2.7%)
**Level of rib-head dislocation**	
Apex-1	10 (18.9%)
Apex	23 (43.4%)
Apex + 1	19 (35.8%)
Apex + 2	1 (1.9%)
**Type of dislocated rib head**	
Single level	23 (62.2%)
Double levels	12 (32.4%)
Triple levels	2 (5.4%)
**Fusion segments**	11.8 ± 1.7 (range, 9–14)
**Implant density** (%)	62 ± 11.1 (range, 42.8–83.3)
**Ratio of laminar hook** (%)	9.8 ± 12.9 (range, 0–37.5)

### Surgical outcomes

The spinal implant constructs were all pedicle screws for 21 patients and hybrid hook-screw constructs for 16 patients. The main thoracic curve and the focal kyphosis improved significantly after surgery from 68.5 ± 15.9° and 60.4 ± 14.8° to 39.1 ± 16.5° and 35.0 ± 13.6, respectively (*p* < 0.001) (Table 2).

**Table 2:** Comparison of spinal deformity parameters and rib-head-related parameters pre- and post-operatively ($\overline{x }$±s)

	**Pre-op**	**Post-op**	***P value***
Main curve (°)	68.5 ± 15.9	39.1 ± 16.5	< 0.001*
Focal kyphosis (°)	60.4 ± 14.8	35.0 ± 13.6	< 0.001*
Intraspinal rib length (mm)	9.2 ± 3.0	4.1 ± 3.9	< 0.001*
DRCSSC (mm)	14.9 ± 5.0	19.7 ± 5.8	< 0.001*
Rib-vertebrae angle (°)	62.6 ± 16.2	53.3 ± 15.3	0.014*
Vertebral-sternum angle (°)	29.7 ± 12.1	20.7 ± 10.1	< 0.001*
Vertebral translation (mm)	22.3 ± 8.2	9.7 ± 5.7	< 0.001*
Height of main curve (mm)	90.0 ± 23.6	103.2 ± 27.0	0.116

^*^Statistically significant (*p* value < 0.05)

*DRCSSC* Distance between the rib head tip and the most concave spot of the spinal canal

On the CT scans, the preoperative rib-head-related parameters including intraspinal rib length, DRCSSC, rib-vertebrae angle and vertebral-sternum angle were 9.2 ± 3.0 mm, 14.9 ± 5.0 mm, 62.6 ± 16.2° and 29.7 ± 12.1°, respectively. After surgery, they were all significantly corrected to 4.1 ± 3.9 mm, 19.7 ± 5.8 mm, 53.3 ± 15.3° and 20.7 ± 10.1 mm, respectively (*p* < 0.05) (Table 2).

The vertebral translation was significantly smaller after correction surgery (22.3 ± 8.2 mm vs. 9.7 ± 5.7 mm, *p* < 0.001). The height of main curve was improved from 90.0 ± 23.6 mm preoperatively to 103.2 ± 27.0 mm postoperatively, though no statistically significant difference was detected (*p* = 0.116) (Table 2).

Screw/hook placement at 37 vertebrae with RHDs were detected in 27 patients, including 6 screws in the convexity, 22 screws in the concavity and 9 hooks in the convexity. Thus, these 37 dislocated ribs were assigned into the screw/hook group. The remaining 16 dislocated ribs without screw/hook insertion at corresponding vertebrae consisted of non-screw/hook group (Table 3).

**Table 3:** Distributions of the patients, the dislocated rib heads and the screw/hook types in screw/hook group and non-screw/hook group

	**Single level**	**Double levels**	**Triple levels**	**Total**
**Screw/hook group**				
Patients	15	10	2	27
Dislocated rib heads	15	20	6	41
Screws	12	14	2	28
convexity	3	3	0	6
concavity	9	11	2	22
Hooks	3	4	2	9
**Non-screw/hook group**				
Patients	8	2	0	10
Dislocated rib heads	8	4	0	12

As to accuracy of pedicle screw placement at corresponding RHD levels, 9 pedicle screws were detected to be malpositioned, including 7 breaching medially and 2 breaching laterally of the pedicles. Further, among the 9 malpositioned pedicle screws, 5 were on the convex side and 4 were on the concave side of the main curve. Except for one screw that breached the lateral vertebral body on the concavity, all the remaining 27 pedicle screws (50.9%, 27/53) at corresponding RHD levels were inserted into the corresponding vertebral bodies. No neurological deficit was complained after surgery.

### Comparisons of the correction rates between the screw/hook group and the non-screw/hook group

No significant difference was detected in terms of correction rate of focal kyphosis, DRCSSC and spinal height between the two groups (Table 4). The correction rates of IRL, RVA and vertebral-sternum angle were significantly higher in screw/hook group than non-screw/hook group (63.1 ± 31.3% vs. 30.1 ± 20.7%, *p* = 0.008; 17.6 ± 9.7% vs. 7.2 ± 3.6%, *p* = 0.006; 24.4 ± 11.0% vs. 11.9 ± 11.3%, *p* = 0.007). Similarly, on the standing posteroanterior radiographs, the correction rates of main curve and vertebral translation were significantly higher in screw/hook group than non-screw/hook group (58.7 ± 16.0% vs. 30.9 ± 12.4%, *p* = 0.003; 61.8 ± 18.8% vs. 35.1 ± 16.6%, *p* = 0.001) (Table 4).

**Table 4:** Comparisons of correction rates of different deformity parameters in screw/hook group and non-screw/hook group ($\overline{x }$±s)

Correction rate (%)	Screw/hook group (*n* = 37)	Non-screw/hook group (*n* = 16)	*P* value
Main curve (%)	58.7 ± 16.0	30.9 ± 12.4	0.003*
Focal kyphosis (%)	49.8 ± 13.4	26.9 ± 10.4	0.148
Intraspinal rib length (%)	63.1 ± 31.3	30.1 ± 20.7	0.008*
DRCSSC (%)	-40.0 ± 26.1	-27.3 ± 17.6	0.203
Rib-vertebrae angle (%)	17.6 ± 9.7	7.2 ± 3.6	0.006*
Vertebral-sternum angle (%)	24.4 ± 11.0	11.9 ± 11.3	0.007*
Vertebral translation (%)	61.8 ± 18.8	35.1 ± 16.6	0.001*
Height of main curve (%)	16.3 ± 9.2	11.9 ± 7.7	0.363

^*^Statistically significant (*p* value < 0.05)

*DRCSSC* Distance between the rib head tip and the most concave spot of the spinal canal

### Linear regression analysis

Multiple linear regression analysis revealed that the correction rates of the main curve, rib-vertebrae angle and vertebral translation were significantly correlated with the correction rate of intraspinal rib length (β = 0.389, 1.869 and 0.939, respectively; *p* = 0.019, 0.002 and 0.001, respectively) (Table 5). The equation was accordingly established as follow: correction rate of intraspinal rib length = -0.510 + 0.389*correction rate of main curve + 1.869*correction rate of rib-vertebrae angle + 0.939* correction rate of vertebral translation.

**Table 5:** The relationship between intraspinal rib length and other parameters via multiple linear regression analysis

Correction rate (%)	β	95% CI	*P *value
Main curve	0.389	0.078–0.701	0.019*
DRCSSC	-0.075	-0.247–0.097	0.360
Rib-vertebrae angle	1.869	0.825–2.912	0.002*
Vertebral-sternum angle	0.196	-0.117–0.509	0.070
Vertebral translation	0.939	0.496–1.382	0.001*
Height of main curve	0.430	-0.160–1.021	0.139

^*^Statistically significant (*p* value < 0.05)

*DRCSSC* Distance between the rib head tip and the most concave spot of the spinal canal

## Discussion

Multiple challenges of deformity correction have been well documented in treating the complicated DS-NF1, including the management of dislocated rib head penetrating into spinal canal. The neurologic status and the severity of intra-spinal occupation by rib head are the pivotal factors that determine the treatment strategy for this condition [8]. No reports have been addressed the effectiveness of apical fixation on treating RHD. The present study, for the first time, comprehensively assessed and confirmed the benefits that screw/hook placement at vertebra with RHD could improve the extraction degree of the penetrated rib head from the spinal canal in DS-NF1.

Previously, resecting the dislocated rib head should be aggressively performed when the following two aspects were indicated [5, 7, 8]. First, the spinal cord being compressed by rib head was detected by T2-weighted MR images [8]. Second, painful rib hump by palpation induced symptoms of radicular neuralgia or limb weakness [7]. After posterior laminectomy and decompression, penetrated rib head could be directly visualized and resected [6]. However, this intra-spinal procedure increased bleeding and operative time, which may bring new risk of neurological complications [4]. Moreover, adhesion between the rib head and the dural sac of the spinal cord should be carefully evaluated, so as to avoid aggravating the nerve damage by violent traction. Accordingly, Mukhtar et al. proposed a ‘left in situ’ strategy as an alternative when the excision of rib head could not be accomplished directly via posterior approach [6]. This concept involved preservation of rib head and removal of a 5-cm segment of rib’s lever arm, which could be helpful for avoiding the interference and traction of the spinal cord and meanwhile eliminating the injury of the ‘leverage’ effect of the rib tip on the spinal cord. Besides, removal of periosteum completely was a necessary preventative method for the regeneration of ribs and reconstruction of the continuity between the osteotomy ends [6]. The remained rib head might get reshaped over time [6]. However, no validated follow-up data was reported currently.

The above-mentioned management strategies were apposite for patients with existing or impending neurological impairment, while for patients who were neurologically intact, whether or not to resect the dislocated rib head was still in controversial. The current mainstream perspective was more inclined to retain the dislocated rib head to avoid interfering the spinal cord, tearing the dural sac, and reducing the bleeding and operative time [5, 10]. Mao et al. suggested that more correction of vertebrae translation and restoration of normal RVA was beneficial for reducing the invasion of the rib head dislocated into the spinal canal [5]. Cai et al. indicated that this phenomenon could be attributed to the laxity or dislocation of the costovertebral articulations, which allowed withdrawal and pullout of the penetrated rib being possible when the corresponding vertebrae was dragged to the concavity following corrective maneuvers [17]. This implied a passive mechanism that superior deformity correction, which was more or less correlated with higher implant density [18], was beneficial for extracting the dislocated rib head from spinal canal. On the other hand, the degree of retraction of dislocated rib head could theoretically be further improved if the vertebrae with RHD were anchored and dragged back to midline with well derotation, serving as an active mechanism. Therefore, screw/hook insertion at vertebra with RHD was supposed to be an ideal method retracting the penetrated intracanal rib head in DS-NF1.

Technically, screw insertion at the pedicle was relatively challenging compared with hook placement at the lamina. The dystrophic and thin pedicles brought about significant challenge of reliable pedicle screw insertion, high frequency of screw malposition [19] and high risk of cord injury. Aside from mutable screw trajectory, low bone mineral density in NF-1 might also contribute to the poor biomechanical properties of the pedicle-screw at these levels [19]. All these technical difficulties and the attendant risks rendered the apical dystrophic spinal segments a screw-forbidden zone in most circumstance. Currently, no quantitative data was available supporting that the screw/hook placement at dystrophic pedicle was beneficial and should be tried with utmost effort in case of RHD with impending neurological deficits.

The result of the present study showed a rate of 50.9% (27/53) for pedicle screw placement in the corresponding dystrophic vertebrae with dislocated rib head. And the correction rates of Cobb angle, IRL, VT and the RVA in the screw/hook group were significantly higher than those in the non-screw/hook group. Further, more corrections of Cobb angle, VT and the RVA contributed significantly to the correction of IRL through multiple linear regression analysis. The above results confirmed that after screw/hook placement combining with higher corrections of Cobb angle, VT and the RVA at vertebrae with RHD, the three-dimensional spatial relationship between rib head and spinal canal could be changed via translational traction and derotational withdrawal. This was helpful for maximizing the degree of spontaneous withdrawal of dislocated intracanal rib head. Accordingly, if possible, the screw/hook placement at vertebrae with RHD should be aggressively advocated. With the extensive clinical application of O-arm navigation, the precise screw placement will be further increased [19]. This was confirmed by Jin et al. that the accuracy of pedicle screw insertion in apical region was increased from 67% with free-hand technique to 79% with O-arm-based navigation technique [19]. Aside from the immediate postoperative withdrawal, the increased spinal stability provided by rigid screw/hook constructs at apical area was also beneficial for preventing progression of RHD and the attendant risks of cord injury [10], all of which were supportive of rib head retaining strategy in neurologically intact DS-NF1 patients.

The clinical relevance of the current study is that despite being risky and challenging, screw/hook placement at dystrophic vertebrae with RHD was beneficial for spontaneous retracions of the intracanal rib head. For patients without neurological symptoms, preoperative pedicle, lamina and vertebral body morphology at the corresponding level of RHD should be carefully evaluated, which is crucial for assessing the possibility and risk in apical fixation. Navigation-based technique can be used to improve the accuracy of pedicle screw placement. If the pedicle at corresponding level of RHD is not a good recipient to well contain the pedicle screw, the ‘in–out-in’ technique is applicable [20]. Otherwise, the lamina hook should be considered as an alternative when pedicle screw insertion was failed. For extreme cases, if the lamina is too thin and osteoporotic to anchor the hook, the segment corresponding to dislocated rib head can be left without apical fixation. In this situation, increasing the implant density in the adjacent segments and improving the curve flexibility using Grade 2 osteotomy are indirect ways to improve the withdrawal of rib head by increasing the corrections of Cobb angle, vertebral translation and rotation. During all these procedures, intraoperative neurophysiological monitoring is indispensable. Lastly, we would like to emphasize that the outcome of this management strategy was highly correlated with the surgeon’s experience and operative technique, and should be carried out meticulously. These aforementioned knowledges are essentially instructional and would assist in treating patients with this condition.

The limitations of this study should be addressed. First, CT evaluations at the long-term follow-up were not available currently. Thus, whether or not the correction of the extracted rib head would be lost due to the loss of main curve correction, formation of pseudarthrosis or failure of internal fixation could not be precisely elucidated. Second, it was impossible to tell the difference between screw and hook insertion on degree of withdrawal of rib head due to the limited sample size.

## Conclusions

Screw/hook insertion at the apical vertebra with RHD is beneficial for retracting the corresponding rib head from spinal canal in DS-NF1. This effectiveness is dependent more on higher corrections of VT and RVA, and could be enhanced by posterior release using posterior column osteotomy.

## Data Availability

The datasets generated and/or analyzed during the current study are not publicly available but are available from the corresponding author on reasonable request.

## Abbreviations

RHD: Rib head dislocation
DS-NF1: Dystrophic scoliosis of Type I neurofibromatosis
VT: Vertebral translation
IRL: Intraspinal rib length
RVA: Rib-vertebral angle
